# The Analgesic Effect of Electroencephalographic Neurofeedback for People With Chronic Pain: Protocol for a Systematic Review and Meta-analysis

**DOI:** 10.2196/22821

**Published:** 2020-10-08

**Authors:** Negin Hesam-Shariati, Wei-Ju Chang, James H McAuley, Andrew Booth, Zina Trost, Chin-Teng Lin, Toby Newton-John, Sylvia M Gustin

**Affiliations:** 1 Centre for Pain IMPACT Neuroscience Research Australia Sydney Australia; 2 School of Psychology University of New South Wales Sydney Australia; 3 School of Medical Sciences University of New South Wales Sydney Australia; 4 School of Health and Related Research University of Sheffield Sheffield United Kingdom; 5 Department of Physical Medicine and Rehabilitation Virginia Commonwealth University Richmond, VA United States; 6 Australian Artificial Intelligence Institute Faculty of Engineering and Information Technology University of Technology Sydney Sydney Australia; 7 Graduate School of Health University of Technology Sydney Sydney Australia

**Keywords:** EEG neurofeedback, chronic pain, meta-analysis, systematic review

## Abstract

**Background:**

Chronic pain is a global health problem, affecting around 1 in 5 individuals in the general population. The understanding of the key role of functional brain alterations in the generation of chronic pain has led researchers to focus on pain treatments that target brain activity. Electroencephalographic neurofeedback attempts to modulate the power of maladaptive electroencephalography frequency powers to decrease chronic pain. Although several studies have provided promising evidence, the effect of electroencephalographic neurofeedback on chronic pain is uncertain.

**Objective:**

This systematic review aims to synthesize the evidence from randomized controlled trials to evaluate the analgesic effect of electroencephalographic neurofeedback. In addition, we will synthesize the findings of nonrandomized studies in a narrative review.

**Methods:**

We will apply the search strategy in 5 electronic databases (Cochrane Central Register of Controlled Trials, MEDLINE, EMBASE, PsycInfo, and CINAHL) for published studies and in clinical trial registries for completed unpublished studies. We will include studies that used electroencephalographic neurofeedback as an intervention for people with chronic pain. Risk-of-bias tools will be used to assess methodological quality of the included studies. We will include randomized controlled trials if they have compared electroencephalographic neurofeedback with any other intervention or placebo control. The data from randomized controlled trials will be aggregated to perform a meta-analysis for quantitative synthesis. The primary outcome measure is pain intensity assessed by self-report scales. Secondary outcome measures include depressive symptoms, anxiety symptoms, and sleep quality measured by self-reported questionnaires. We will investigate the studies for additional outcomes addressing adverse effects and resting-state electroencephalography analysis. Additionally, all types of nonrandomized studies will be included for a narrative synthesis. The intended and unintended effects of nonrandomized studies will be extracted and summarized in a descriptive table.

**Results:**

Ethics approval is not required for a systematic review, as there will be no patient involvement. The search for this systematic review commenced in July 2020, and we expect to publish the findings in early 2021.

**Conclusions:**

This systematic review will provide recommendations for researchers and health professionals, as well as people with chronic pain, about the evidence for the analgesic effect of electroencephalographic neurofeedback.

**Trial Registration:**

International Prospective Register of Systematic Reviews (PROSPERO) CRD42020177608; https://www.crd.york.ac.uk/PROSPERO/display_record.php?RecordID=177608

**International Registered Report Identifier (IRRID):**

PRR1-10.2196/22821

## Introduction

### Background

Chronic pain is estimated to affect up to 50% of the adult population [1,2], and 10% to 20% experience clinically significant chronic pain [3]. Chronic pain is defined as ongoing or recurrent pain, lasting for at least three months [4,5]. It is often associated with functional limitations and psychological distress [4,6], resulting in a decreased health-related quality of life [7,8]. Chronic pain may result from an ongoing pathology (eg, cancer), damage to the central nervous system (eg, stroke and spinal cord injury) or peripheral nervous system (eg, diabetic neuropathy), tissue degeneration (eg, arthritis), and other pain syndromes with unknown pathologies (eg, fibromyalgia and complex regional pain syndrome).

The understanding of the critical role of maladaptive functional brain changes in the development and maintenance of chronic pain has led researchers to focus on pain treatments that aim to modulate brain activity [9,10]. Previously, neurosurgical methods, such as cordotomy and thalamotomy, were considered to be effective in the control of abnormal brain activity, such as increased theta frequency power, resulting in a significant pain reduction [11,12]. However, these types of surgery are costly, highly invasive, and associated with major complications such as cognitive impairment. In the past few decades, noninvasive brain stimulation techniques including transcranial direct current stimulation, cranial electrotherapy stimulation, and repetitive transcranial magnetic stimulation have been used to reduce pain by aiming to alter the maladaptive brain activity associated with chronic pain. However, there is insufficient evidence to support the efficacy of these approaches on chronic pain [13,14]. More recently, electroencephalographic (EEG) neurofeedback using brain-computer interface technology has been developed to target the maladaptive brain activity underlying chronic pain [2,15].

### Description of EEG Neurofeedback

The goal of EEG neurofeedback is to modulate the targeted maladaptive EEG frequency powers to decrease chronic pain [2,15,16]. Surface EEG is recorded from 1 or more electrode sites, depending on the specific pain condition, often from the sensorimotor cortex [17]. The targeted frequency powers are extracted and processed in real time, then presented to the individual as visual or auditory feedback, or both [16,18]. For example, it has been shown that individuals with chronic neuropathic pain have increased theta and reduced alpha frequency power compared with healthy individuals without chronic pain [19,20]. In this case, EEG neurofeedback is used to suppress theta and reinforce alpha frequency power [2,16]. Using this EEG neurofeedback protocol, individuals can learn to regulate their abnormal brain activity in a way that reduces their chronic pain [2,16].

### Previous Reviews and Rationale

Previous systematic reviews about the effect of EEG neurofeedback on chronic pain have mainly focused on specific pain conditions such as fibromyalgia [21,22] or cancer-related pain [23]. The results of these systematic reviews were inconclusive due to the limited data. While a recent review found a medium effect size of pain reduction favoring neurofeedback interventions in chronic pain, it included studies using functional magnetic resonance imaging-based neurofeedback [24]. Combining the results for 2 different methodologies makes it difficult to evaluate the analgesic effect of a specific intervention.

Our planned systematic review will cover all forms of chronic pain and include only EEG-based neurofeedback interventions to increase the likelihood of conclusive evidence about the analgesic effect of EEG neurofeedback. Although inclusion of a wide variety of pain conditions will increase the heterogeneity of the pooled data, the larger sample size will substantially improve the meta-analytic power. In order to distinguish the effect of EEG neurofeedback on different chronic pain types, such as neuropathic and nonneuropathic pain, we will conduct a subgroup analysis (see the Subgroup and Sensitivity Analysis subsection below). Review findings will inform researchers and health professionals, as well as people with chronic pain, about the analgesic effect of EEG neurofeedback. In addition, this review can help to identify any gaps in previous studies and provide direction for future research.

### Objectives

The primary objective of this systematic review is to evaluate the evidence for the analgesic effect of EEG neurofeedback for people with chronic pain. The secondary objective is to investigate the effect of EEG neurofeedback on depressive symptoms, anxiety symptoms, and sleep quality. Further, as an additional objective of this systematic review, we will include the reports of adverse events and resting-state EEG analysis for a narrative review.

## Methods

### Review Registration

This systematic review protocol is prepared according to the Preferred Reporting Items for Systematic Reviews and Meta-Analysis Protocols (PRISMA-P) 2015 guidelines [25,26]. Multimedia Appendix 1 is the PRISMA-P checklist. This systematic review is registered in the International Prospective Register of Systematic Reviews with registration number CRD42020177608.

### Types of Participants

We will include studies of participants with chronic pain, defined as persistent or recurrent pain for more than 3 months [4]. There will be no restriction on age or sex of the participants in the included studies.

### Types of Intervention

We will include studies that investigate the analgesic effect of EEG neurofeedback for people with chronic pain, regardless of the number and duration of intervention sessions, the EEG neurofeedback protocol, and the targeted brain region.

### Types of Outcome Measures

The *primary* outcome measure is pain intensity. Pain intensity may be assessed using a self-report rating scale such as the visual analog scale or the numeric rating scale. Studies that used other scales will also be included.

The *secondary* outcome measures are depressive symptoms, anxiety symptoms, and sleep quality. Depressive symptoms and anxiety symptoms may be measured by self-report questionnaires such as the Beck Depression Inventory or Beck Anxiety Inventory, or the Hamilton Depression Rating Scale or Hamilton Anxiety Rating Scale. Sleep quality may be assessed using the Medical Outcomes Study Sleep Scale or the Pittsburgh Sleep Quality Index. Studies that have used other assessments will not be excluded.

We will include studies that have assessed the primary or secondary outcome measures, or both, on at least two occasions, one before or at the beginning of the intervention and one close to or at the end of the intervention. Further, we will include additional outcome measures for the narrative review. For example, reports of any adverse effects will be included as well as the results of the resting-state EEG analysis comparing pre- versus postintervention.

### Types of Studies

We will include randomized controlled trials (RCTs) if they have compared EEG neurofeedback with no treatment or any other intervention, including sham control, waitlist control, or usual care. Nonrandomized studies, defined as “any quantitative study estimating the effectiveness of an intervention that does not use randomisation to allocate subjects to comparison groups” [27], will be included for a narrative review. Comparative nonrandomized studies (eg, cross-sectional designs and controlled cohort studies) will be used to address intended effects, and noncomparative studies (eg, case reports and case series) will be reported for corroborating evidence and adverse effects. All studies must have used EEG neurofeedback as an intervention for people with chronic pain. We will exclude studies that involved the following: (1) individuals experiencing pain for less than 3 months; (2) healthy individuals with experimentally induced pain; and (3) any other intervention in conjunction with EEG neurofeedback.

### Search Strategy

To identify the eligible studies, we will search 5 electronic bibliographic databases for published studies: (1) Cochrane Central Register of Controlled Trials (CENTRAL), (2) MEDLINE, EMBASE, and PsycInfo via Ovid, and (3) CINAHL via EBSCO.

Additionally, we will search the following clinical trial registries for completed unpublished studies: (1) ClinicalTrials.gov, (2) EU Clinical Trials Register, (3) Australia New Zealand Clinical Trials Registry, and (4) World Health Organization International Clinical Trials Registry Platform (ICTRP).

Search strategies will be established using Medical Subject Headings (MeSH) and related text words. We will use a combination of different keywords for chronic pain and EEG neurofeedback intervention to identify relevant literature. The search strategies will be tailored to each database. Multimedia Appendix 2 shows the search strategy according to Ovid search syntax. There will be no restriction on the publication period, but only articles in English language will be included. In addition, we will check the reference lists of the eligible studies and relevant review articles to include any missed but relevant published studies. While the review is in progress, citation searching for forward citation of recent studies and citation alerts (eg, on Google Scholar) on included studies will be used to identify new studies as they appear. The searches will be rerun prior to the final analysis and further retrieved studies will be included.

### Study Selection

We will use EndNote X9 (Clarivate Analytics) reference software to store, organize, and manage all the search results and ensure an efficient study selection process by removing the duplicate records. Two reviewers will independently evaluate the title and abstract of all studies identified through the search against the inclusion and exclusion criteria. Any disagreement between the individual judgments will be resolved by an additional reviewer. The screening process will be conducted in Covidence (Veritas Health Innovation Ltd), which is systematic review management software. The full text of the selected studies will then be retrieved. In the case of trial registrations, the full text is defined as all associated files and information. If the reviewer is uncertain about the eligibility of any study, the full text will be obtained for further information. An additional reviewer will be consulted, should there be any uncertainty or disagreement of the eligibility of studies. Disagreement on study eligibility will be resolved through consensus. Excluded studies and the reasons for exclusion will be recorded and documented.

### Data Extraction

We will pilot test a customized data extraction spreadsheet on 2 studies relevant to this review, and then use it to extract data from the eligible studies. Two reviewers will independently extract the data from the final list of studies. The disagreements in the extracted data will be resolved through discussion with an additional reviewer. The following information will be extracted from the eligible studies.

We will extract data on *study characteristics*, including the study design, country, and setting of the study.

We will extract *participant* data on diagnosis, age, sex, duration of pain, comorbidities, and the number of participants allocated in each intervention group. The primary and secondary outcome measures at baseline (ie, before or at the beginning of the intervention) will also be extracted (mean and measure of variability).

We will extract *intervention* data on EEG neurofeedback protocols including the targeted frequency bands, the targeted brain region, the duration of each session, the number of sessions, and the duration of the interventions. Data on the details of the comparative intervention (ie, type, dosage, frequency) in each individual study will also be extracted.

We will extract data on the type of *outcome measures* used to assess the primary and secondary outcomes, the time points from baseline to the end of interventions, and follow-ups. The postintervention assessments will be categorized into 3 groups: short-term for less than 1 week, mid-term for 1 to 6 weeks, and long-term for more than 6 weeks for follow-up assessments.

We will extract *results* of the primary and secondary outcome measures at a time point close to or at the end of the interventions, or the changes in outcome measures from baseline for each intervention group. If a study used more than 1 outcome measure of pain intensity, we will select and extract only a single measure, prioritizing them in the following order: 100-mm/10-cm visual analog scale, 11-point numeric rating scale (0 = no pain, 10 = the worst pain imaginable), and then pain intensity rating from composite measures or other scales [28].

For the secondary outcome measures, we will extract scores from each of the questionnaires for depressive symptoms, anxiety symptoms, and sleep quality if the studies used more than 1 questionnaire. We will also extract the number of participants who stopped receiving the treatment due to a rare or adverse event in each intervention group.

If data are missing, we will contact the authors of the studies a maximum of 3 times, after which we will consider the data to be irretrievable.

### Study Quality and Risk of Bias

Study quality and risk of bias will be assessed by 2 independent reviewers using the first version of the Cochrane Risk of Bias (RoB 1.0) tool for RCTs [29] and the Cochrane Risk of Bias in Non-Randomised Studies - of Interventions (ROBINS-I) tool for nonrandomized studies [30]. Additionally, the quality of noncomparative studies (eg, case reports and case series) will be assessed using the Joanna Briggs Institute critical appraisal tools [31]. The inconsistencies will be resolved by an additional reviewer.

We will use the Cochrane RoB 1.0 tool to assess the study-level risk of bias for 5 domains: selection, performance, detection, attrition, and reporting bias [29]. We will use the ROBINS-I tool to assess the risk of bias for studies that have not used randomization for intervention allocations, such as cohort studies and cross-sectional designs. The risk-of-bias assessment using this tool covers 7 domains: confounding and participants’ selection (preintervention), intervention classification (during intervention), and deviations, missing data, measurements, and selection of reported results (postintervention) [30]. The ROBINS-I tool includes signaling questions to provide easier judgments for each domain, as well as an overall risk-of-bias assessment. We will use the Joanna Briggs Institute critical appraisal checklists [31] for case reports and case series to assess the study-level risk of bias.

### Data Synthesis

We will not combine the data extracted from RCTs and nonrandomized studies for a quantitative synthesis. The distinctions between various types of nonrandomized studies and RCTs make it methodologically indefensible to pool the results in a meta-analysis [32]. The extracted outcomes data from RCTs will be quantitatively synthesized by a meta-analysis method using R (R version 4.0.0; R Foundation for Statistical Computing) software. The population and intervention from at least two RCTs must be sufficiently similar to perform a meta-analysis. Indeed, the level of consistency and appropriateness of RCTs is key to justify pooling the results in a meta-analysis [33].

We will convert the primary and secondary outcome data to a 0- to 100-point scale (mean and standard deviation) [28]. In numerical or continuous scales, the score value is divided by the range of scale, and then multiplied by 100. For example, for a 0 to 20 scale, the score value is divided by 20 and multiplied by 100. Likert scales will be treated as numerical scales, because the scores for Likert-type questions can be summed and presented as a final scale score. Additionally, in categorical scales, the lowest value will be assigned to be 0, and then 1 additional point for each category of severity. For example, none = 0, mild = 1, moderate = 2, and high = 3. Then, these values will be treated like numerical scales.

The relative treatment effects of the compared interventions (eg, EEG neurofeedback vs control) on the outcome measures will be estimated using weighted mean difference with 95% confidence intervals [28]. We will use a threshold of 10 points on the 0- to 100-point scale to clarify the minimal clinically important effect of EEG neurofeedback on pain intensity [34]. Since a cutoff threshold has not been established for converted 0 to 100 points of the secondary outcomes, we will adopt a 10-point threshold as the clinically meaningful change for depressive symptoms, anxiety symptoms, and sleep quality.

In recognition of the likely heterogeneity of the chronic pain population and the EEG neurofeedback methodology, we will use a random-effects meta-analysis. We will assess the heterogeneity of the study population and intervention using the χ^2^ test and estimate the degree of heterogeneity using the *I*^2^ statistic. The heterogeneity is considered significant when *P*<.1 and when *I*^2^≥50%. A subgroup analysis will be performed when significant heterogeneity is present (see Subgroup and Sensitivity Analysis subsection).

We will conduct a narrative synthesis to provide additional information about EEG neurofeedback as an intervention including adverse effects. The data and methodology for the great variety of nonrandomized designs are usually not sufficiently similar to be pooled in a meta-analysis; thus, we will use a narrative approach for these studies [35]. Narrative methods of synthesis include classification of evidence from diverse studies, data reduction, data display, comparison, and conclusion [36]. The findings from the nonrandomized studies will be described and summarized in an extraction table using techniques of narrative synthesis.

### Quality of Evidence

We will use the Grading of Recommendations Assessment, Development and Evaluation (GRADE) approach [37] to grade the certainty of evidence and the strength of recommendations at the outcome level. For example, the GRADE rating will be applied to the outcome of interest to estimate the certainty of the intervention effect. There are 4 levels of certainty within the GRADE approach: very low, low, moderate, and high. The level of certainty of evidence can be downgraded for the following reasons.

#### Risk of Bias

The rating will be downgraded by 2 levels if there is a high risk of bias for more than 25% and less than 50% of the included studies’ participants. It will be 1 grade down if more than 50% of participants are from high risk-of-bias studies [38].

#### Imprecision

The rating will be downgraded by 1 level if the total number of participants is less than 400 for continuous data and less than 300 for dichotomous data [39].

#### Inconsistency

The rating will be downgraded by 1 level if significant heterogeneity is identified (*P*<.1) [40].

#### Indirectness

This domain will not be considered because the inclusion criteria of this review ensures a specific population and outcome interest [41].

#### Publication Bias

The rating will be downgraded by 1 level if a publication bias is detected using visual and statistical assessments [42].

### Subgroup and Sensitivity Analysis

Where heterogeneity is identified (*P*<.1), we will conduct subgroup analysis according to the type of chronic pain and the study population age through preplanned analysis: (1) neuropathic pain versus nonneuropathic pain: neuropathic pain is defined as “pain caused by a lesion or disease of the somatosensory nervous system” [43], and nonneuropathic pain includes all other chronic pain conditions; (2) adults versus adolescents or children: studies including adults over 18 years old compared with studies with individuals under 18 years old.

Further, depending on the variability of RCTs, we will conduct a sensitivity analysis to assess the impact of excluding studies with high risk of bias.

## Results

This review will not require any ethics approval, as there will be no patient involvement in the conduct, reporting, and interpretation of the review. The search for this systematic review commenced in July 2020, and we will disseminate the findings as soon as they are available, expected by early 2021.

## Discussion

This protocol describes the methodology of a systematic review and meta-analysis to aggregate the evidence for analgesic effects of EEG neurofeedback for people with chronic pain. In addition to including RCTs for a meta-analysis, we will supplement the review by a narrative synthesis of nonrandomized comparative designs for intended effects and noncomparative designs for corroborating evidence and adverse effects.

The heterogeneity of the chronic pain population and the variety of EEG neurofeedback methodology might restrict the opportunities for meta-analysis and interpretation of results. However, preplanned subgroup analyses based on the pain conditions and patients’ age groups will help to address the issue of population heterogeneity.

We will report the methodology and results of this review according to the PRISMA guidelines [44]. The findings will provide an evaluation of both the intended and adverse effects of EEG neurofeedback interventions. Given the debilitating impact of chronic pain on people’s quality of life, this systematic review will provide recommendations for researchers, health care professionals, and people with chronic pain about the evidence for the analgesic effect of EEG neurofeedback.

## Appendix

Multimedia Appendix 1 Preferred Reporting Items for Systematic Reviews and Meta-Analysis Protocols (PRISMA-P) checklist.

Multimedia Appendix 2 Search strategy.

## Abbreviations

CENTRAL: Cochrane Central Register of Controlled Trials
EEG: electroencephalography
GRADE: Grading of Recommendations Assessment, Development and Evaluation
ICTRP: International Clinical Trials Registry Platform
MeSH: Medical Subject Headings
PRISMA-P: Preferred Reporting Items for Systematic Reviews and Meta-Analysis Protocols
RCT: randomized controlled trial
RoB 1.0: Risk of Bias version 1.0
ROBINS-I: Risk of Bias in Non-Randomised Studies - of Interventions
