# Supplementary material for: The Analgesic Effect of Electroencephalographic Neurofeedback for People With Chronic Pain: Protocol for a Systematic Review and Meta-analysis
Source: JMIR Res Protoc. 2020 Oct 8;9(10):e22821. doi: 10.2196/22821 (PMC7582146; doi:10.2196/22821)
Supplement: Multimedia Appendix 2 [file resprot_v9i10e22821_app2.pdf]

Search strategy through OVID for MEDLINE, Embase, and PsycInfo

(Restricted to Humans and English Language)

MEDLINE (OVID), Embase (OVID), PsycINFO (OVID)

1 exp pain/

2 ((chronic* or back or musculoskel* or intractabl* or neuropath* or phantom limb or fantom limb or neck or myofasc* or temporomandib* joint* or temperomandib* joint* or tempromandib* joint* or central or post*stroke or complex or regional or spinal cord) adj4 pain*).tw.

3 (sciatica or back-ache or back*ache or lumbago or fibromyalg* or (trigemin* adj2 neuralg*) or (herp* adj2 neuralg*) or (diabet* adj2 neuropath*) or (reflex adj4 dystroph*) or (sudeck* adj2 atroph*) or causalg* or whip-lash or whip*lash or whiplash or polymyalg* or (failed back adj4 surg*) or (failed back adj4 syndrome*)).tw.

4 or/1-3

5 exp electroencephalography/

6 (electroencephalogra* or EEG* or quantitative EEG or QEEG*).tw.

7 or/5-6

8 exp neurofeedback/

9 (neuro*feedback or biofeedback or bio*feedback or neuromodulat* or neuro*modulat* or neurotherapy or neuro*therapy or BCI* or brain*computer interface or BMI* or brain*machine interface).tw.

10 or/8-9

11 4 and 7 and 10
